# Exploring the Predictive Potential of Physiological Measures of Human Thermal Strain in Outdoor Environments in Hot and Humid Areas in Summer—A Case Study of Shanghai, China

**DOI:** 10.3390/ijerph20065017

**Published:** 2023-03-12

**Authors:** Zefeng Lian, Binyi Liu, Robert D. Brown

**Affiliations:** 1 Department of Landscape Architecture, Suzhou University of Science and Technology, Suzhou 215011, China; 2 Gold Mantis School of Architecture, SooChow University, Suzhou 215005, China; 3 College of Architecture and Urban Planning, Tongji University, Shanghai 200092, China; 4 Department of Landscape Architecture and Urban Planning, Texas A&M University, College Station, TX 77843, USA

**Keywords:** thermal strain prediction, heart rate variability, non-invasive measure, outdoor environment, hot and humid climate area, cumulative link mixed model

## Abstract

Whenever people spend time outdoors during hot weather, they are putting themselves in potentially stressful situations. Being able to predict whether a person is overheating can be critical in preventing heat-health issues. There is a clear relationship between body core temperature and heat health. However, measuring body core temperature is expensive. Identifying a non-invasive measure that could indicate a person’s thermal strain would be valuable. This study investigated five physiological measures as possible surrogates: finger mean skin temperature (FSKT), finger maximum skin temperature (FMSKT), skin conductance level (SCL), heart rate (HR), and heart rate variability (HRV). Furthermore, they were compared against the results of participants’ subjective thermal sensation and thermal comfort in a range of hot microclimatic conditions in a hot and humid climate. Results showed that except for SCL, each of the other four physiological measures had a positive significant relationship with thermal sensation, but a negative relationship with thermal comfort. Furthermore, through testing by cumulative link mixed models, HRV was found to be the most suitable surrogate for predicting thermal sensation and thermal comfort through a simple, non-invasive measure in outdoor environment in summer in a hot and humid area. This study highlights the method for predicting human thermal strain and contributes to improve the public health and well-being of urban dwellers in outdoor environments.

## 1. Introduction

Humans are quickly becoming an urban species, with most people living in cities. In some places such as the US, the proportion of urban population is greater than 80% [1]. However, the way cities have been developed in the past has made them hotter than the surroundings [2]. This exacerbates the global trend toward a warmer climate and puts human health at risk. Staying in outdoor environments in summer is potentially dangerous for people, particularly during heat waves [3]. Examples of thermal stress situations include kids playing football, the elderly being vulnerable and unable to recognize heat stroke symptoms, people dying during heat waves, dangers of overheated microclimates, etc. [4].

Thus, predicting human thermal strain to avoid causing heat-related disease in outdoor environments becomes crucial. The main trends to investigate and predict thermal strain, both outdoors and indoors, consist of two ways: one is through the on-site questionnaire survey and the other employs the empirical equation by fitting questionnaire and energy budget model, such as physiological equivalent temperature (PET), wet bulb globe temperature index (WBGT), Universal Thermal Comfort Index (UTCI), etc. [5]. However, those widely used energy budget models do not fully consider individual characteristics. With the advance of wearable devices and the marching to smart cities, using physiological parameters measured by wearable devices to investigate thermal strain has become the frontier of this field [6].

Body core temperature indicating the temperature of the internal organs is the most accurate physiological measure which reflects human thermal strain based on the human thermoregulation mechanism [7]. However, measuring body core temperature (internal) is expensive. Thus, it is necessary and valuable to find a simple, non-invasive surrogate to determine when people are too hot and in danger of heat-related diseases, such as heat stroke.

### 1.1. Widely Used Non-Invasive Physiological Measures for Thermal Strain

Physiological responses of a human body to thermal environments have been broadly investigated in lots of research of both outdoor and indoor environments, concerning temperature regulation mechanism, autonomic nervous system, cardiovascular system, etc. Several physiological measures have been widely recognized as the representations of human thermal strain, such as finger skin temperature [8], skin conductance [9], heart rate [10], and heart rate variability [11].

#### 1.1.1. Skin Temperature

Skin temperature is the thermal status of the outer skin layer of a human body. Unlike the body core temperature, which is maintained in a narrow range, the fluctuation of skin temperature varied in a larger threshold, which resulted from thermal stress which may by induced by the change in body core temperature as well as other potent factors such as the change in air temperature and evaporation of sweating. For example, finger skin temperature has been used as one of the main indicators of physiological thermal strain and plays an essential role in human thermoregulation [12].

When a human body is suffering from thermal stress, the widening of blood vessels as well as the congestion of capillaries speed the heat loss up [13], resulting in an increase of skin temperature.

Researchers have employed skin temperature to investigate thermal strain and found they are significantly correlative. For example, the skin temperature was significantly lower by 1.1 degrees Celsius in a tree shadow compared with in open space [14]. There is a significant correlation between thermal strain and skin temperature of seven local body parts in different indoor thermal environments [15]. Moreover, the accuracy of thermal comfort prediction was over 95% with the skin temperature of the human face as an independent variable in an indoor experiment that employed infrared thermography [16]. The mean skin temperature and its gradience were two critical factors for subjects’ thermal sensation [17].

#### 1.1.2. Skin Conductance Level

Skin conductance level represents the conductivity between two skin surface points, determined by sweat secretion activity [18]. Based on physiological mechanisms, sweat secretion results from the enhancement of sweat gland activity induced by stimulus, such as stress, etc. It is recognized as an indicator of sympathetic nerve activity [19] and sweat activity in human thermoregulation [20].

Therefore, skin conductance is widely employed in the research of psychological stimuli, as well as thermal strain [21]. Wang and Hu found sweat activity was linearly related to thermal sensation vote [22]. Nicola et al. proposed that skin conductance performed well in predicting thermal comfort with a r^2^ higher than 0.71 during walking [9]. 

Body core temperature rises when a human body is in a hot microclimate [23]. At the same time, a negative feedback mechanism is activated. Then, the thermoregulation mechanism generates neural signals through the back of the hypothalamus and finally stimulates the sweat glands to secrete sweat, which takes the excessive body heat away through evaporation [24]. Therefore, people would feel stuffy in high humidity environment because sweat on the skin is hard to evaporate from the body’s surface.

#### 1.1.3. Heart Rate

Heart rate refers to the number of heartbeats per unit time [25]. Heart rate fluctuates with thermal stress [26], as well as physical activity and psychological change.

When a human body suffers thermal stress in a microclimate, it must dissipate excessive heat outwards by increasing the skin blood flow, as well as the sweating. So, only by increasing the frequency of heart contraction and relaxation, i.e., increasing the heart rate, can homeostasis be maintained. For example, Choi et al. found that by comparing the cool chamber (18 °C~20 °C) and the warm chamber (25 °C~27 °C), the heart rate of men in the latter was significantly higher [27].

#### 1.1.4. Heart Variability

Heart rate variability (HRV) represents the degree of variation between the two successive R-waves of sinus beats signal [28]. It refers to the regularity of the heartbeat, as the more regular the pattern is, the lower the HRV, and vice versa.

HRV reflects the activity and balance of the human autonomic nervous system (ANS), including the sympathetic nervous system (SNS) and the parasympathetic nervous system (PNS) [29]. When the SNS is activated, it is associated with the perception of arousal or stress, which increases heart rate, represented by the lower frequency (LF) power [30]. In contrast, the activation of the PNS is related to relaxation, which causes a decrease in heart rate, represented by the high frequency (HF) power [30]. Therefore, LF/HF can reflect the balance of the ANS, which has been recognized as a widely used indicator of HRV [31].

Negative feedback is activated when a human body experiences thermal stress to keep body core temperature in a narrow range through thermo-regulation mechanism. At this time, the SNS is activated to promote sweating and vasoconstriction to dissipate heat away from the body, along with the increase in heart rate [32]. Therefore, LF/HF would increase, and HRV would decrease. When people feel thermally comfortable, a human body consumes less oxygen and nutrients than thermally uncomfortable, resulting in gentler breathing. Therefore, the PNS and SNS are more balanced, represented by the decreased LF/HF and increased HRV.

HRV is proved to be highly correlated with thermal strain in lots of studies. For example, Liu et al. found LF/HF was significantly higher when subjects felt thermally uncomfortable than thermally comfortable [33]. Similar evidence had also been reported by other research [34,35]. Furthermore, Cong et al. proposed that cold exposure would decrease SNS activity and increase skin temperature, represented by a lower LF/HF [36]. Yang et al. proved LF/HF went up along with the increase of thermal sensation. Particularly, when it approaches 1, subjects would feel neutral or slightly warm [37]. Unlike those results found in indoor settings, Liu et al. compared the differences in participants’ physiological responses between two microclimates and indicated that LF/HF under a tree was significantly higher than in the open space [14].

### 1.2. Research Gap and Aim

Although those research studies mentioned above show evidence of the relationship between physiological measures and human thermal strain, research gaps still exist. Firstly, most of the research was conducted in an indoor environment, and a limited number of studies were in outdoor spaces [14,17]. Unlike the steady-state condition of an indoor space, an outdoor microclimate is non-steady, namely dynamic [17,38]. Therefore, exploring the relationship between physiological measures and thermal strain in urban outdoor spaces characterized by dynamics is urgent under the background of climate change, as well as validating the existing evidence found indoors.

Secondly, most research has just investigated the relationship between one physiological measures and thermal strain [37]. Several studies compared the predictive potential of more than two measures to thermal comfort indoors [39]. However, there is no research that compared the significance level of correlation and predictive potential of skin temperature, skin conductance, heart rate, and heart rate variability to thermal strain.

Thirdly, the regression method widely used to explore the prediction of physiological parameters to thermal strain was linear [9,22,36] and curve-linear [35], which have been proved to be not the most suitable [40,41,42]. Logistic regression was found to be more appropriate [5,17], taking the participant as a random factor.

This study aimed to find a simple, non-invasive way to determine when people are too hot and in danger of thermal stress in outdoor environments. Using Shanghai as a case study, and by conducting an on-site experiments and questionnaires in three urban squares in summer, we investigated and compared the correlation and predictive potential between each of the five widely used physiological measures as mentioned above (finger mean skin temperature, finger maximum skin temperature, skin conductance level, heart rate, and heart rate variability) and thermal strain (thermal sensation and thermal comfort) to determine if any could be used as the best surrogate of body core temperature measurement, due to its expensiveness to predict thermal strain in urban outdoor space in hot and humid climate areas in summer.

## 2. Materials and Methods

### 2.1. Experiment Site

The experiment was conducted in Shanghai, China, in three urban squares with three days each, between 20 July and 21 August 2018, that are considered to be the typical day of summer and supposed to be hot and sunny according to weather forecast. The exact dates of the measurements in each square and the corresponding outdoor weather conditions are shown in Table S1. Shanghai belongs to the subtropical humid climate zone, with a typical characteristic of cold winter and hot summer, located between 120°52′ E to 122°12′ E and 30°40′ N to 31°53′ N. Shanghai experiences the lowest air temperature near 0 °C in winter, and the highest air temperature close to 40 °C in summer, with a prevailing wind direction of southeast and an annual average relative humidity of 68%.

**Figure 1:**
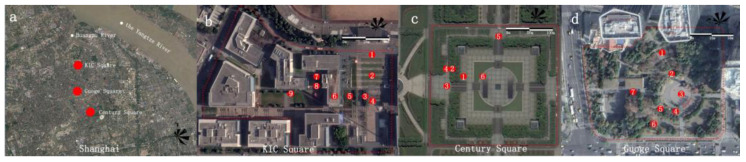
Experiment sites and spots. (**a**) Shanghai and locations of the three squares: (**b**) KIC square, (**c**) Century square, and (**d**) Guoge square. Numbers in the sub-figures indicates the experiment spots.

We chose Knowledge and Innovation Community (KIC) square (Figure 1b), Century square (Figure 1c), and Guoge square (Figure 1d) as experiment sites based on the criteria of spatial diversity, elevation variation, aspect ratio, and sky view factor of each square which could result in a wide range of microclimates, after surveying most of urban square in Shanghai downtown area. Furthermore, 22 spots were selected based on different space attributes to conduct experiments with 6, 7, and 9 at Century square, Guoge square, and KIC square, respectively. Detailed information about each spot is shown in Table S2.

### 2.2. Participants

Seventy healthy adults without cardiovascular disorders or skin disease, including twenty-six females and forty-four males, were recruited to participate in the experiment through social media. Table 1 shows the demographic information of age, weight, height, body mass index (BMI), and clothing thermal resistance (clo).

All participants had lived in Shanghai for at least three years, which indicated they would have been acclimatized [43].

Participants were informed of the experiment procedure and were required to observe the following precautions: avoid staying up late, drinking alcohol, or taking drugs before the experiment. They were also required to meet the experiment dress code to keep the similar clothing thermal resistance. 

Participants were randomly assigned to the experiment time slot of each square, which resulted in 27 for Century square, 25 for KIC square, and 18 for Guoge square, based on their available day.

### 2.3. Microclimate and Human Physiological Measures

Watchdog 2000 series (Spectrum Technologies, Inc., Aurora, IL, USA, 2021), a portable weather station (Table S3), was employed to measure the microclimate condition of each spot at the same time on experiment days. It monitored microclimate parameters, including air temperature (Ta), relative humidity (Rh), solar radiation (Sr), wind speed (Ws), and wind direction with a logging interval of 1 min. The watchdog was installed on a tripod, keeping it near 1.5 m above the ground that made the height of sensors close to a participant’s heart and head. 

A structured questionnaire was designed to survey participants’ thermal strain. The first part was demographic information, including name, age, weight, height, and dress. The second part, consistent with ASHARE Standard 55 [44], included a thermal sensation vote (TSV) with a nine-point Likert scale, and an outdoor thermal comfort vote (OTC) with a five-point Likert scale (Figure S1).

ErgoLAB “Human-Machine-Environment” synchronization platform (Kingfar Inc. Beijing, China, 2014), which has been validated in related research [14,45], was employed to measure participants’ physiological response in each spot with different microclimates. It consists of three wearable wireless sensors to monitor different signals (finger skin temperature, skin conductance level, heart rate, and heart rate variability) and a data processing platform. Table S4 shows the accuracy and range of each sensor.

### 2.4. Experimental Protocol

**Figure 2:**
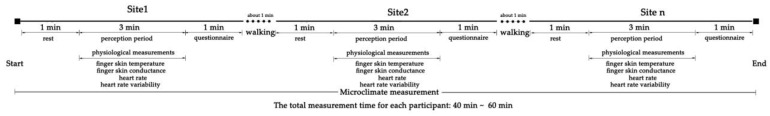
Experiment protocol.

Figure 2 shows the experimental protocol. At the beginning of each experiment day, Watchdog weather stations were assembled and started to continuously log microclimate data at each spot during the test.

Secondly, after participants arrived, they were reminded of the experiment protocol again and signed the informed consent. Then, researchers helped them wear wireless physiological sensors. Three sensors were attached on the index finger (to measure skin temperature), middle and fourth fingers (to measure skin conductance), and the earlobe (to measure heart rate and heart rate variability), respectively.

Thirdly, each round of the test included two participants. After preparation, they walked at a uniform pace to the first spot, then rested for 1 min, that has been proved to be suitable in the pretest, to minimize the physiological disturbance by walking. Next, there was a 3 min thermal stress perception period with a standing posture. At the beginning of this period, wireless wearable sensors start to record the physiological signals simultaneously and stop when it ends. After this period, participants needed to fill out the questionnaire in 1 min. Then, participants walked to the next spot and repeated the same process until all the test spots were finished. Each participant takes about 40 min~60 min to finish the entire experiment.

The sequence of experiment spots was designed based on the sky view factor to avoid participants from being overexposed to the sun for a long time (Supplementary Material Table S2).

### 2.5. Statistical Analysis

The descriptive analysis of microclimate data was conducted in SPSS 20.0 (IBM Corp, Armonk, NY, USA, 2011). FSKT, FMSKT, SCL, HR, and HRV were processed on the ErgoLAB platform. FSKT and SCL were both processed by moving average filter.

HR and HRV were first filtered by baseline de-noise, white de-noise, high-pass de-noise, and low-pass de-noise. Then, ectopics were replaced to analyze LF/HF.

Correlation analysis between each physiological measure and thermal strain was conducted using the ‘rmcorr’ package in R 3.5.0 (R Core Team, Vienna, Austria, 2018), based on repeated measurement correlation, with participants’ ID as a random factor.

Furthermore, cumulative link mixed model (CLMM) was used to fit the quantitative model between each physiological measure and thermal strain, with the former as the independent variable and the latter as the dependent variable. CLMM had been testified in related research to fit the model with ordinal data as dependent variable [46,47]. Thus, it can meet the ordinal data attribute of thermal strain designed based on the Likert scale, as well as dig the latent correlation of repeated measurements induced by the within-subject experiment design, which has been neglected by previous research. 

We selected log-likelihood and Akaike information criterion (AIC) to compare the predictive potential of physiological measure to the thermal strain of each model. Studies have shown that the better model was both with the lower AIC [48] and the higher log-likelihood [49].

The significant difference level in this research was set at *p* < 0.05. 

## 3. Results

### 3.1. Microclimate and Thermal Strain during Experiment

Microclimate parameters, including Ta, Sr, Rh, and Ws, were logged simultaneously at each spot from 7:30 a.m. to 17:30 p.m. in the experiment days of each square. Table 2 shows the values of microclimate parameters and participants’ thermal strain which is represented by TSV and OTC during the experiment. According to the minimum (Min), maximum (Max), and standard deviation (SD), it can be seen that the microclimate condition was wide-ranging, representing the variability of space configuration among those spots. Moreover, it is worthy to notice that wind speed was low during the experiment.

For TSV, participants voted from neutral (0) to too hot (+4). For OTC, it ranged from too uncomfortable (−2) to very comfortable (+2), which means the samples were well distributed. In contrast, the overall values of TSV and OTC during the experiment were warm (1.83 ± 1.26) and neutral (−0.23 ± 0.88).

### 3.2. Thermal Physiology Response

Participants’ physiological response induced by the surrounding microclimate were monitored and logged during the perception period at each spot using three wireless wearable sensors. Data were analyzed through the Ergo-Lab platform and SPSS. After removing those recordings with abnormal and missing data, Table 3 shows the numbers of the sample, Min, Max, mean, and SD of each physiological measure at each square and the overall. First, each measure has 354 samples, with 83, 155, and 116 for KIC, Century, and Guoge square, respectively. Second, based on the Min and Max, participants’ physiological responses varied a lot during the experiment, representing the abundant microclimate condition, consistent with Section 3.1.

Based on mean and SD of the overall value, FSKT mostly ranged from 34.09 °C to 36.47 °C, while it is 34.48 °C~37.24 °C for FMSKT, with the highest value of 41.66 °C. There is a big difference between the Min and Max of SCL, with a mean value of 5.21 μs. For heat rate, participants in a specific microclimate scenario have an extreme value of 135 bpm. Moreover, the mean and SD indicated that participants’ heart rates were within the normal domain in most microclimate conditions. Lastly, for LF/HF, its majority ranged from 0.47~2.43, with a highest record at 5.99.

### 3.3. Physiological Measures and Thermal Strain

#### 3.3.1. Correlation between Physiological Measures and Thermal Strain

Because the experiment was designed based on within-subject test, the data were not independent. So, the repeated measures correlation statistical method was employed to test the significant correlation between each physiological measure and thermal strain [50], taking participants into account, with the ‘rmcorr’ package in R. Table 4 shows that except for SCL, the others all had significant correlations with OTC and TSV. Furthermore, all were negative with OTC, which means when these physiological measures increase, OTC will decrease; the situation for TSV is inverse.

Moreover, among these correlation coefficients of physiological measures for OTC, LF/HF was the highest with −0.34, followed by FSKT and FMSKT, with both being 0.31. For TSV, the overall pattern was similar, but correlation coefficients of all measures were higher than that of OTC. The highest correlation coefficient was LF/HF with 0.48, followed by FSKT and FMSKT. Lastly, HR shows the lowest correlation coefficient with TSV and OTC, with 0.12 and 0.16, respectively, compared with other parameters.

#### 3.3.2. The Best Fitted Model for Predictive Potential of Physiological Measure to Thermal Strain

In this study, three aspects require us to carefully choose the suitable analysis algorithm that meets the data attribute and structure to demonstrate the latent information of the database. Firstly, unlike the random survey adopted by most research studies, this experiment was designed based on the within-subject test. Thus, recordings of each subject are nested, namely not independent. Secondly, TSV and OTC, as the dependent variables, were designed based on the Likert scale (which is ordinal data). Recent studies have questioned whether linear regression can demonstrate the exact quantitative relationship with ordinal data as the dependent variable and proposed that logistic regression may be more suitable [40,41]. Thirdly, it is better to treat the subject as a random factor (random intercept) in the regression to minimize the effect of individual differences on the model accuracy, because of the individual difference which induced the variance of physiological response between subjects under thermal strain, especially SCL shown in Table 3.

Therefore, linear regression turned out to be not the most suitable for this research, and cumulative link mixed models [51], employed CLMM function from “ordinal Package” in R (R Core Team, 2016), were selected to regress the quantitative relationship between each physiological measure and thermal strain. CLMM had been testified in related research to fit the model with ordinal data as dependent variable [46,47]. Thus, it can meet the ordinal data attribute of thermal strain designed based on the Likert scale, as well as dig the latent correlation of repeated measurements induced by the within-subject experiment design, which has been neglected by previous research.

All models of each physiological measure and thermal strain are listed in Table 5, showing dependent variable, model number (Model no.), syntax, independent variable, and random effect. Thus, ten cumulative linked mixed models were fitted with each physiological measure as independent variable, as well as controlling participants’ ID as the random effect.

Table 5 shows the models’ information of these ten CLMM, listing log-likelihood, AIC, and significant difference between each model and above (Pr).

Firstly, according to Pr, results show that the predictive potential of each model is significantly different from the others, with *p* < 0.001. Secondly, for all CLMM of OTC, the best model is M1.4, with the lowest AIC of 854.70, and the highest log likelihood of −421.35, followed by M1.1, M1.5, and M1.2, with the last one of M1.3. This indicates that LF/HF has the most predictive potential for OTC, followed by FMSKT, HR, and FSKT, while SCL is the last.

Thirdly, for all CLMM of TSV, the best model is M2.4, with the lowest AIC of 1040.80, and the highest log likelihood of −499.85. The second one is M2.1, followed by M2.2, and M2.5, while M2.3 is the last with the highest AIC of 1093.5, and the lowest log likelihood of −540.75. This demonstrates that LF/HF has the best predictive potential for TSV, followed by FMSKT, FSKT, and HR, while SCL is the last. 

Lastly, comparing all predictive potential of physiological measures for OTC and TSV shows that all the models of OTC perform better than that of TSV, with an apparent difference in AIC and log-likelihood.

## 4. Discussion

### 4.1. Microclimate Condition and Its Effects of Thermal Strain and Thermal Physiology Response

The microclimate condition and participants’ physiological responses in this experiment were rich with a wide range value of microclimate parameters, thermal strain, as well as physiological measures, which provides a sound database for the further analysis, as shown in Table 2 and Table 3. Especially, the maximum of four physiological measures in Table 3 indicates that participants experienced thermal stress during the experiment. This can be proved by comparing the skin temperature response reported in Tianjin [17] as well as Hall [52], the threshold of heart rate proposed by Moran et al. [53], the typical range of skin conductance by Cacioppo [54], and the norms of LF/HF by Malike et al. [55] and Shaffer et al. [29]. So, exploring the suitable physiological measures to predict thermal strain in the outdoor environment is required and meaningful to make our city more comfortable, and protecting people from heat-related diseases and even death in summer.

In our study, except SCL, the others all have significant correlations to thermal strain. HRV has the most significant correlation with thermal strain. While there are few similar outdoor experiments results that could be compared, several indoor experiments support our finding. For example, Zhu et al. reported a clear pattern of LF/HF to both thermal comfort and thermal sensation [56]. Moreover, Wu Guoshan et al. also found that LF/HF would significantly fluctuate under different thermal stress, especially between neutral and hot environments for mine workers [57].

The correlation coefficient of FSKT to OTC and TSV was lower than HRV, but higher than other physiological measures, with a value of 0.43, which is not so expected high compared to those reported results of indoor experiments [58]. However, our finding is supported by Vanos et al., who presented that the correlation coefficient between skin temperature and actual thermal sensation is 0.32 in outdoor environments [59]. 

There was no significant relationship between skin conductance and thermal strain, which is similar with the results found in Singapore [60]. While it is different to findings of indoor experiments [61], this may because of the excessive sweat that is secreted by sweat glands and induced by the continued stimulus of thermal stress outdoor in summer. Furthermore, the sweat cannot be well-absorbed or evaporated due to the low wind speed during the experiment. This finding indicates that, unlike indoor environments, skin conductance in outdoor environments may not change according to the thermal stress stimulus outdoor because of excessive sweat, especially when wind speed is low. 

It is noted that the correlation coefficients of physiological measures to thermal sensation are all below 0.5, and higher than that to thermal comfort. There may be two reasons that could explain this phenomenon. Firstly, the percentage of physiological measure that can contribute to thermal stress evaluation was only around 50%, according to the previous research [62]. Secondly, based on brain science, the consciousness of thermal sensation is produced in the primary somatic sensory cortex, after it is passed through the thalamus [63,64]. In contrast, related research has proposed that thermal comfort is synthesized in the frontal cortex and the limbic system, after it is passed through the hypothalamus [65], which demonstrates that the neural pathway of thermal comfort is more complex than that of thermal sensation. 

### 4.2. The Best Predictive Potential of Physiological Measures of Human Thermal Strain in Summer

Based on AIC and log-likelihood, Table 6 demonstrates LF/HF is the best physiological measure to predict both thermal comfort and thermal sensation by employing the CLMM function in R. This finding is in agreement with Nkurikiyeyezu et al., who reported HRV could precisely predict thermal state up to 93.7% by machine learning classification algorithms [66]. Moreover, several indoor research results also proved LF/HF is a good indicator for the prediction of thermal strain. For example, Zhu et al. proposed that higher LF/HF indicates thermal discomfort, while low LF/HF presents a more acceptable thermal state [35]. Moreover, Wu et al. found that a participant’s LF/HF is significantly different between hot and neutral environment controlled by indoor climate chamber [57].

This may be because HRV represents the condition of the cardiac system, which is the most important for the human thermal regulation system. The change rate of skin temperature mediated by vasodilation and vasoconstriction, the sweating intensity, and respiration frequency were all supported by the function of the heart, which is represented by HRV. Take vasodilation as an example. When the participant is under thermal stress, as well as body core temperature deviates from the acceptable range, a human body needs to dissipate excessive heat. Thus, more blood will be transported to the skin surface to dissipate heat through radiation and convection. These processes are supported by the firing of sympathetic nerve that increases heart pumping volume, resulting in the increase of low-frequency portion and the change of HRV.

At the same time, sweat glands are activated to secrete sweat thus dissipating the heat through evaporation, due to the vibration of the sympathetic nervous system through the sudomotor. To support and maximize evaporation, the sympathetic nervous system secretes noradrenaline to increase heart rate and cardiac output. Thus, the cooler venous blood could be circulated faster to lower core temperature, as well as to increase the water supply from vessel to sweat gland. This process would also increase the portion of low frequency power of HRV. 

Similarly, the increase of respiration, which is also a path to dissipate excessive body heat, is mediated by high frequency power and needs to be coupled with the increase of cardiovascular system circulation modulated by the sympathetic nervous system through the heart.

After body core temperature falls into the acceptable range, the activity of potential action of sympathetic nerve decreases, resulting in the decrease in low frequency and the increase in HRV. So, HRV is somewhat like a signal amplifier that becomes the most prominent index to reflect the thermoregulation process. Thus, it would have the most predictive potential for thermal strain in the outdoor environment in summer.

An unexpected result comes up that finger maximum skin temperature has a predictive potential, which is just lower than heart rate variability but higher than others. However, we find that few research studies have investigated the predictive potential of maximum skin temperature for thermal strain, but studied the gradient of skin temperature [67]. Only one piece of literature supported our finding and reported that the maximum skin temperature could be used to discriminate thermal comfort and discomfort when it comes to 39 degrees Celsius [68]. We speculate that two reasons would contribute to the result. Firstly, the 3 min thermal perception period in each experiment is a little bit short. Secondly, when the finger skin temperature comes to the maximum, a human body may experience the extreme thermal stress of the perception period, particularly in summer. Furthermore, this feeling of experience might be stored in the specific brain area which is responsible for short-term memory storage, such as the pre-front cortex, hippocampus, and amygdala [69]. 

For skin conductance, interestingly, contrasted to those findings that propose skin conductance level is a sound indicator, from much indoor research related to thermal comfort [61] emotion [70], or landscape perception [71]. In this outdoor research, we found SCL has no predictive potential for thermal strain. There are two reasons that can explain the result. Firstly, excessive sweat was detained on the skin surface, because the activation of the sympathetic nervous system continuously stimulates the sweat gland. At the same time, sweat evaporation was suppressed, due to the low wind speed. So, excessive sweat on the skin surface could not be evaporated nor absorbed, making the measurement of SCL unable to reflect the change in the human body’s thermal state timely. Therefore, we speculated that SCL might have the predictive potential when the wind speed is enough to evaporate sweat from the skin surface or the sweat gland can absorb the excess sweat, which also means SCL would be a good indicator when the human body feels warm but not hot or very hot, such as in spring and autumn.

### 4.3. Limitations and Future Research

There are several limitations of this study that need to be improved in further research. Firstly, due to the lack of body core temperature, the prediction potential difference between HRV and body core temperature is unclear. Future studies can focus on comparing these two indices. Secondly, other physiological measures that might also have predictive potential for thermal strain, such as skin temperature gradient and electroencephalography (EEG), can be taken into consideration in future research. Thirdly, participants of this study were adults. Whether those results are applicable to children and older people needs to be further investigated as an important topic. Fourthly, what contributes to the different predictive potentials of HRV to thermal comfort and thermal sensation needs to be further investigated more deeply from the perspective of neurology and brain science. Moreover, our findings were just found in Shanghai, while they need to be further verified in other places in the hot and humid area, even compared with results from other climate areas.

Lastly, unlike indoor experiments, we cannot eliminate the environment inference such as the sound effects in outdoor environments. Even those experiment sites chosen are places where car horns are prohibited around the surrounding street. Future studies could use other alternative methods to decrease the sound effect on the thermal stress as much as possible.

## 5. Conclusions

This research proposed the research question: which non-invasive physiological measures could be surrogates of body core temperature to determine when people are too hot in an outdoor environment. Taking Shanghai as a case study, we conducted outdoor experiments at three urban squares in summer, with microclimate measurement, thermal stress questionnaire, and physiological measurement for 9 days between 20 July and 21 August 2018. This research investigated the correlation and predictive potential between each of the five physiological measures and thermal strain in the urban outdoor environment in hot and humid climate areas in summer. HRV had the best predictive potential for thermal strain, which can be the best surrogate of body core temperature, followed by finger maximum skin temperature, finger mean skin temperature, and heart rate. Skin conductance was unsuitable for predicting thermal strain outdoor environment in summer. Notwithstanding several limitations, our research has demonstrated that HRV has the potential to be employed to predict thermal strain in hot and humid climate areas in summer.

The applications of the research findings will be beneficial in three aspects. Firstly, HRV could be employed to design real-time outdoor thermal devices in the future, which will adjust forms based on the prediction of people’s thermal strain received from the non-invasive wearable sensors. Secondly, the results will be beneficial to the post-occupancy evaluation of urban design which will generate evidence-based guidelines for planners, designers, as well as policy makers to create a more livable, comfortable, and intelligent urban outdoor environment. Lastly, the results suggest HRV could be the surrogate of core temperature which can be used to protect the health of urban residents, thus improving their well-being, especially in summer.

## Figures and Tables

**Table 1:** Demographics of participants.

Sex	Participants (n)	Age (Years)	Height (m)	Weight (kg)	BMI (Body Mass Index, kg/m^2^)	Clo (Clothing Thermal Resistance)
Male	44	35.90 ± 11.90	1.70 ± 0.10	67.20 ± 7.60	22.70 ± 2.20	0.66 ± 0.15
Female	26	29.20 ± 9.80	1.60 ± 0.10	52.90 ± 6.90	19.90 ± 2.20	0.79 ± 0.16

Note: values are means ± standard deviation.

**Table 2:** Microclimate condition and thermal strain during experiment.

		Ta (°C)	Sr (W/m^2^)	Rh (%)	Ws (m/s)	TSV	OTC
Overall	Min	29.90	9.80	38.90	0.01	0	−2
	Max	38.50	1105.60	74.50	3.16	+4	+2
	Mean ± SD	33.13 ± 1.68	290.36 ± 280.95	55.18 ± 7.32	0.41 ± 0.57	1.83 ± 1.26	−0.23 ± 0.88
KIC	Min	29.90	12.20	39.40	0.01	0	−2
	Max	38.50	1048.60	70.50	3.16	+4	+2
	Mean ± SD	32.77 ± 1.93	338.11 ± 270.71	53.33 ± 7.02	0.73 ± 0.71	2.11 ± 1.33	−0.38 ± 0.99
Century	Min	30.30	9.80	43.50	0.01	0	−2
	Max	37.20	1105.60	68.70	2.22	+4	+2
	Mean ± SD	33.60 ± 1.56	337.51 ± 320.65	56.39 ± 5.65	0.37 ± 0.52	1.92 ± 1.28	−0.29 ± 0.89
Guoge	Min	30.40	17.20	38.90	0.01	0	−2
	Max	36.50	887.60	74.50	2.22	+4	+2
	Mean ± SD	32.76 ± 1.49	193.19 ± 195.39	54.88 ± 9.08	0.24 ± 0.41	1.51 ± 1.12	−0.02 ± 0.73

**Table 3:** Participants’ thermal physiology response during experiment.

		SKT (°C)		SCL (µs)	HR (Bpm)	LF/HF
		FSKT	FMSKT			
Overall	Sample	354	354	354	354	354
	Mean	33.00	33.31	0.01	67.00	0.23
	Max	39.07	41.66	23.96	135.00	5.99
	Mean ± SD	35.28 ± 1.19	35.86 ± 1.38	5.21 ± 4.38	91.12 ± 12.75	1.45 ± 0.98
KIC	Sample	83	83	83	83	83
	Min	33.00	33.31	0.07	70.00	0.23
	Max	39.07	41.66	23.96	135.00	5.30
	Mean ± SD	34.51 ± 1.38	35.30 ± 1.84	2.53 ± 4.21	91.06 ± 13.67	1.45 ± 0.96
Century	Sample	155	155	155	155	155
	Min	33.12	33.76	0.01	70.00	0.27
	Max	38.25	39.97	18.55	124.00	5.99
	Mean ± SD	35.52 ± 1.21	36.12 ± 1.38	4.53 ± 3.77	96.42 ± 11.81	1.74 ± 1.06
Guoge	Sample	116	116	116	116	116
	Min	33.17	33.91	2.21	67	0.24
	Max	36.83	39.22	18.17	109.00	5.06
	Mean ± SD	35.47 ± 0.70	35.91 ± 0.78	8.03 ± 3.63	84.07 ± 9.55	1.05 ± 0.69

**Table 4:** Overall correlation between each physiological measure and thermal strain during experiment tested by repeat measurement correlation method.

Physiological Measure		Thermal Strain	
		OTC	TSV
SKT	FSKT	−0.31 ***	0.42 ***
	FMSKT	−0.31 ***	0.43 ***
SCL		−0.11	0.13
HR		−0.18 *	0.12 *
LF/HF		−0.34 ***	0.48 ***

Notes: * *p* < 0.05, *** *p* < 0.001.

**Table 5:** Overview of models and syntax employed with cumulative link mixed models.

Dependent Variable	Model No.	Syntax	Independent Variable	Random Effect
OTC	M1.1	Clmm (OTC~FMSKT+(1|ID))	FMSKT	ID
	M1.2	Clmm (OTC~FSKT+(1|ID))	FSKT	
	M1.3	Clmm (OTC~SCL+(1|ID))	SCL	
	M1.4	Clmm (OTC~LF/HF+(1|ID))	LF/HF	
	M1.5	Clmm (OTC~HR+(1|ID))	HR	
TSV	M2.1	Clmm (TSV~FMSKT+(1|ID))	FMSKT	
	M2.2	Clmm (TSV~FSKT+(1|ID))	FSKT	
	M2.3	Clmm (TSV~SCL+(1|ID))	SCL	
	M2.4	Clmm (TSV~LF/HF+(1|ID))	LF/HF	
	M2.5	Clmm (TSV~HR+(1|ID))	HR	

**Table 6:** Overview of CLMM models’ performance represented by log likelihood and AIC.

Model No.	Log-Likelihood	AIC	Pr (>Chisq)
M1.1	−428.64	869.28	***
M1.2	−434.17	880.35	***
M1.3	−443.98	899.97	***
M1.4	−421.35	854.70	***
M1.5	−431.31	874.63	***
M2.1	−514.39	1040.8	***
M2.2	−522.99	1058.0	***
M2.3	−540.75	1093.5	***
M2.4	−499.85	1011.7	***
M2.5	−529.15	1070.3	***

Note: Pr column states whether the model performs significantly better than above. *** *p* < 0.001.

## Data Availability

Data are readily available from the corresponding author upon reasonable request.

## Supplementary Materials

The following supporting information can be downloaded at: https://www.mdpi.com/article/10.3390/ijerph20065017/s1, Table S1: Detail experiment information of each square; Table S2: Detail information of each experiment spot and its sequence; Table S3: Portable weather station parameter information; Table S4: Accuracy and range of each sensor of Ergo-Lab parameters; Figure S1: Urban Square Outdoor Thermal Stress Questionnaire.
