# Supplementary material for: Exploring the Predictive Potential of Physiological Measures of Human Thermal Strain in Outdoor Environments in Hot and Humid Areas in Summer—A Case Study of Shanghai, China
Source: Int J Environ Res Public Health. 2023 Mar 12;20(6):5017. doi: 10.3390/ijerph20065017 (PMC10049132; doi:10.3390/ijerph20065017)
Supplement: Supplementary file 1 [file ijerph-20-05017-s001.zip › Table S1.Detail experiment information of each square.pdf]

Table S1. Detail experiment information of each square

Square	Experiment dates	Weather condition	Experiment time
KIC square	July 20, 21, 28, 2018	mostly clear to sunny	7:30 a.m.-17:30 p.m.
Century square	August 4, 5, 6, 2018	mostly clear to sunny	7:30 a.m.-17:30 p.m.
Guoge square	August 10, 11, 15, 2018	mostly clear to sunny	7:30 a.m.-17:30 p.m.
