# Supplementary material for: Exploring the Predictive Potential of Physiological Measures of Human Thermal Strain in Outdoor Environments in Hot and Humid Areas in Summer—A Case Study of Shanghai, China
Source: Int J Environ Res Public Health. 2023 Mar 12;20(6):5017. doi: 10.3390/ijerph20065017 (PMC10049132; doi:10.3390/ijerph20065017)
Supplement: Supplementary file 1 [file ijerph-20-05017-s001.zip › Table S2.Detail information of each experiment spot and its sequence.pdf]

Table S2. Detail information of each experiment spot and its sequence

**Square**					**KIC**								**Century**							**Guoge**		
Spot	1	2	3	4	5	6	7	8	9	1	2	3	4	5	6	1	2	3	4	5	6	7
Sequence	1	2	3	4	5	6	7	8	9	1	2	3	4	5	6	1	2	3	4	5	6	7
SVF	0.95	0.93	0.12	0.89	0.71	0.09	0.23	0.62	0.66	0.91	0.25	0.84	0.15	0.85	0.71	0.49	0.13	0.83	0.20	0.34	0.30	0.47
Ground type	Gr	Gr	La	Gr	Gra	Gra	Gr	WP	Gra	Gr	Gr	Gr	Gr	Gr	Gr	Wb	Wb	Wb	La	Wb	La	Gr
Tope type	Os	Os	Tc	Os	Os	Cw	Ba	Os	Os	Os	Tc	Os	Cw	Os	Os	Tc	Tc	Os	Tc	Tc	Tc	Os

Notes: Gr: Granite; La: Lawn; Gra: Gravel; Wp: Wood platform; Wb: Water permeable brick; Os: Open sky; Tc: Tree canopy; Cw: Concrete wall; Ba: Bamboo.
