# Supplementary material for: Exploring the Predictive Potential of Physiological Measures of Human Thermal Strain in Outdoor Environments in Hot and Humid Areas in Summer—A Case Study of Shanghai, China
Source: Int J Environ Res Public Health. 2023 Mar 12;20(6):5017. doi: 10.3390/ijerph20065017 (PMC10049132; doi:10.3390/ijerph20065017)
Supplement: Supplementary file 1 [file ijerph-20-05017-s001.zip › Table S3. Portable weather station parameter information.pdf]

Table S3. Portable weather station parameter information

Equipment	Parameter	Range	Accuracy
Watchdog 2000 Series Weather Station	Wind speed	0.3 ~ 241 km/h	±3 km/h
	Wind direction	0° ~ 360°	±4°
	Air temperature	-40°C ~ 125°C	±0.4°C
	Relative humidity	0% ~ 100%	±2%
	Solar radiation	1 W/m^2 ~ 1500 W/m^2	±5%
