# Supplementary material for: Exploring the Predictive Potential of Physiological Measures of Human Thermal Strain in Outdoor Environments in Hot and Humid Areas in Summer—A Case Study of Shanghai, China
Source: Int J Environ Res Public Health. 2023 Mar 12;20(6):5017. doi: 10.3390/ijerph20065017 (PMC10049132; doi:10.3390/ijerph20065017)
Supplement: Supplementary file 1 [file ijerph-20-05017-s001.zip › Table S4. Accuracy and range of each sensor of Ergo-Lab parameters.pdf]

Table S4. Accuracy and range of each sensor of Ergo-Lab parameters.

Equipment	Wearable wireless device	Range	Accuracy
Ergo-Lab "Human-Machine-Environment" synchronization platform	Skin temperature sensor	10 °C ~ 60 °C	±0.1°C
	Skin conductance sensor	0 μs ~ 30 μs	±0.1μs
	Photoplethysmography sensor	25 Bpm ~ 240 Bpm	1 Bpm
