# Supplementary figures and images for: Exploring the Predictive Potential of Physiological Measures of Human Thermal Strain in Outdoor Environments in Hot and Humid Areas in Summer—A Case Study of Shanghai, China
Source: Int J Environ Res Public Health. 2023 Mar 12;20(6):5017. doi: 10.3390/ijerph20065017 (PMC10049132; doi:10.3390/ijerph20065017)

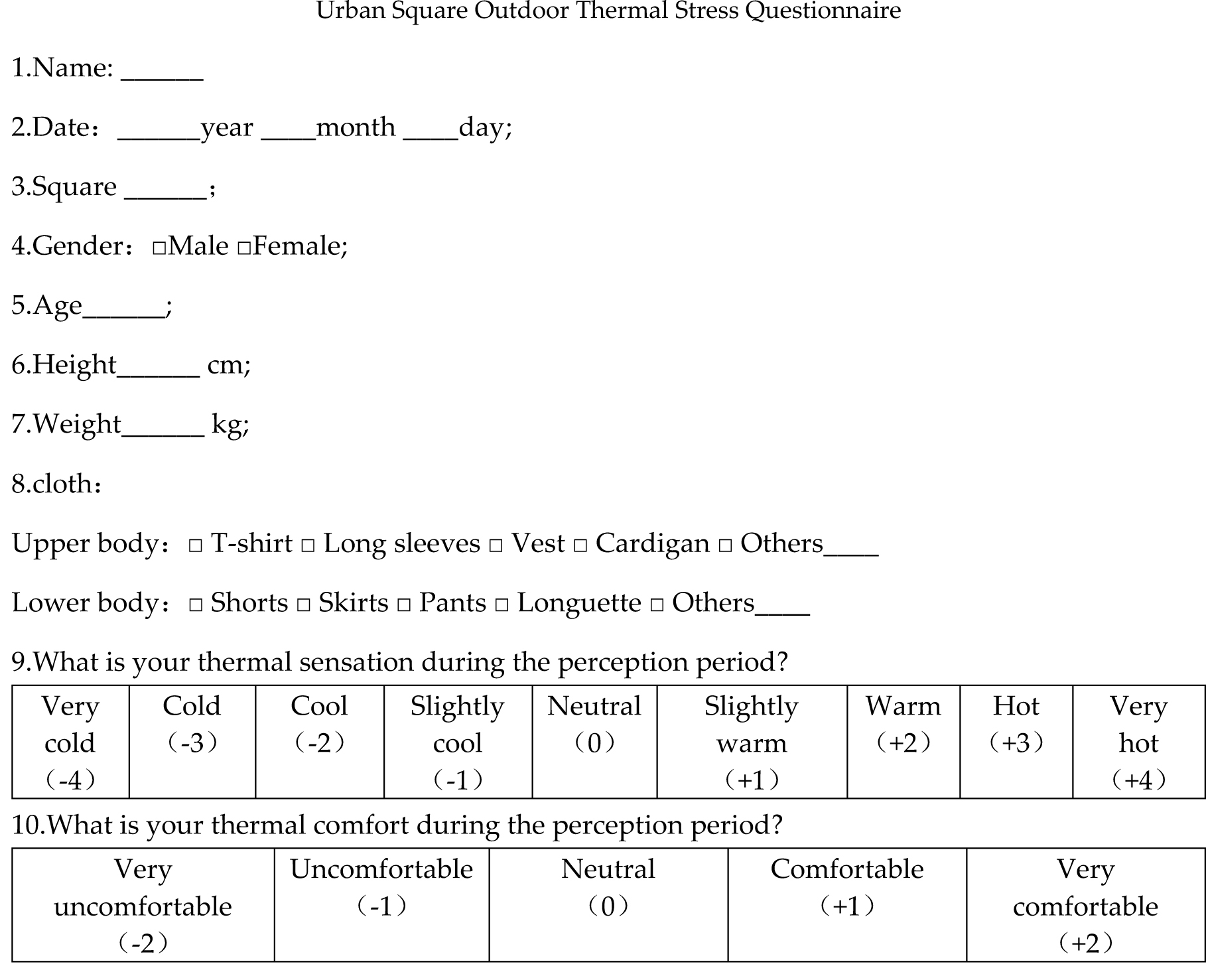

Supplement: Supplementary file 1 [file ijerph-20-05017-s001.zip › Figure S1.Urban Square Outdoor Thermal Stress Questionnaire.jpg]
